# Impact of socioeconomic deprivation in patients undergoing elective surgical resection for colon cancer

**DOI:** 10.1093/bjsopen/zrag014

**Published:** 2026-03-23

**Authors:** Sophie M Tait, Lucia Chung, Paul G Horgan, Campbell S D Roxburgh, Donald C McMillan, Allan M Golder

**Affiliations:** Academic Unit of Surgery, Glasgow Royal Infirmary, Glasgow, UK; Medical Veterinary and Life Sciences and School of Cancer Sciences, University of Glasgow, Glasgow, UK; Medical Veterinary and Life Sciences and School of Cancer Sciences, University of Glasgow, Glasgow, UK; Inverclyde Royal Hospital, Greenock, UK; Academic Unit of Surgery, Glasgow Royal Infirmary, Glasgow, UK; Medical Veterinary and Life Sciences and School of Cancer Sciences, University of Glasgow, Glasgow, UK; Academic Unit of Surgery, Glasgow Royal Infirmary, Glasgow, UK; Medical Veterinary and Life Sciences and School of Cancer Sciences, University of Glasgow, Glasgow, UK; Academic Unit of Surgery, Glasgow Royal Infirmary, Glasgow, UK; Medical Veterinary and Life Sciences and School of Cancer Sciences, University of Glasgow, Glasgow, UK; Academic Unit of Surgery, Glasgow Royal Infirmary, Glasgow, UK; Medical Veterinary and Life Sciences and School of Cancer Sciences, University of Glasgow, Glasgow, UK

**Keywords:** colorectal cancer, short-term survival, deprivation index, cancer mortality, colorectal surgery

## Abstract

**Background:**

Colon cancer and socioeconomic deprivation (SED) are associated with adverse outcomes. This study examined correlations between clinicopathological variables and both SED and survival in tumour node metastasis (TNM) I–III and III cohorts.

**Methods:**

Patients undergoing elective curative resection for TNM I–III colon cancer were identified from the West of Scotland cancer registry. The primary outcome of interest was the association between SED (defined using the Scottish Index of Multiple Deprivation (SIMD); SIMD 1 = most deprived; SIMD 5 = least deprived), short-term (30- and 90-day mortality), mid-term (3-year overall (OS) and cancer-specific (CSS) survival). Secondary outcomes compared SED, the administration of adjuvant chemotherapy and significant tumour and clinical factors (overall and in TNM III patients). Multivariable analyses were conducted to correlate these findings with survival.

**Results:**

A total of 2264 patients were included in the study (790 TNM III). Overall, there was no significant difference between SIMD 1 and 5 in 30-day mortality (2.3 *versus* 1.8%, respectively; *P* = 0.480) and 90-day mortality (3.2 *versus* 2.0%, respectively; *P* = 0.616). OS was lower in SIMD 1 than 5 (83 *versus* 86%; *P* = 0.008), as was CSS (90 *versus* 92%; *P* = 0.024). There was no significant association between SIMD and the receipt of chemotherapy (29.4% *versus* 34.7%, *P* = 0.152) or any tumour factors. Compared with SIMD 5 patients, SIMD 1 patients had a higher American Society of Anesthesiologists (ASA) grade (*P* < 0.001), more current smokers (17.5 *versus* 4.0%; *P* < 0.001), an RCS Charlson Score > 3 (6.6 *versus* 4.3%; *P* < 0.001), obesity (36.0 *versus* 22.7%; *P* < 0.001), and modified Glasgow Prognostic Score (mGPS) = 2 (18.5 *versus* 14.2%; *P* = 0.007). Multivariable analysis confirmed the association with ASA (odds ratio (OR) 1.70; 95% confidence interval (c.i.) 1.31 to 2.20; *P* < 0.001), smoking (OR 1.59; 95% c.i. 1.24 to 2.03; *P* < 0.001), and body mass index (BMI) (OR 1.23; c.i. 1.01 to 1.50; *P* = 0.045). Similar associations were seen among TNM III patients, although SIMD 1 (*versus* 5) patients were less likely to commence adjuvant chemotherapy (59.4 *versus* 73.0%; *P* < 0.10).

**Conclusion:**

Overall, SIMD 1 patients had worse OS in both the both TNM I–III and III cohorts, with co-morbidity and lifestyle factors most likely being responsible.

## Teaser

Socioeconomic deprivation is associated with host factors but not tumour factors, and this is likely the reason for adverse survival in deprived populations.

## Introduction

Colorectal cancer is a significant healthcare concern worldwide being the third most common cancer, with 1.9 million cases diagnosed in 2020^1^. Risk factors for the development of colorectal cancer are multifactorial, but socioeconomic deprivation (SED) remains a significant influencing factor in both the presentation and outcomes of colorectal cancer^2–6^.

SED encompasses multiple complex and inter-related factors that culminate in a lack of social, economic, and health-based resources, thus making it difficult to identify a single responsible factor for poor outcomes. Although SED is easy to define, assumptions of homogeneity within populations by SED scores remain a significant issue when undertaking research into this field, making comparisons between studies difficult and leading to a paucity of higher-level and correlation studies.

The West of Scotland comprises a diverse westernized population containing some of the most deprived areas in Scotland^7^. The Scottish Index of Multiple Deprivation (SIMD) was developed to stratify SED specific to the Scottish population^8^. SIMD is an area-based scoring system using census data. It comprises seven different domains, namely employment, income, health, crime, housing, education, and access to services, and was developed from the Index of Multiple Deprivation score^9^.

Previous studies have predominantly examined SED in relation to combined patient cohorts of colon and rectal cancer, including the association with incidence^5^ and survival^2^, but colon and rectal cancers are increasingly being considered separate disease entities, also due to different therapeutics approaches. The centralization of services, the introduction of minimally invasive surgery, and the rollout of bowel cancer screening programmes have improved outcomes^10–12^ over the past 20 years. However, patients with tumour node metastasis staging system (TNM) III disease are a subgroup that can benefit most from improvements in surgical technique and oncological management. TNM III patients are more likely to have adverse tumour biology and more extensive nodal disease, but still have a potential for cure when compared to other TNM stages. Furthermore, there are potential lifestyle interventions that may benefit these patients^(13)^. Indeed, the multifactorial landscape of SED has changed over time. Adverse lifestyle factors are commonly associated with deprived populations. Obesity was historically associated with affluence but now disproportionately affects deprived populations^14^. Despite smoking cessation campaigns, smoking remains prevalent, particularly within deprived populations^15–17^. Smoking and obesity are both associated with SED and are recognized risk factors for colon cancer. A recent systematic review (unpubl. obs Tait SM, Chung L, Horgan PG, Roxburgh CSD, McMillan DC, Golder AM) highlighted the ongoing link between deprived populations and adverse short-term and long-term outcomes. Therefore, the present study aimed to assess these interactions and their relationship with survival outcomes in a cohort of patients undergoing elective surgery with curative intent for TNM I–III colon cancer and in a further subgroup analysis of patients with TNM III colon cancer.

## Methods

### Study design and setting

A retrospective review of the West of Scotland Colorectal Cancer Managed Clinical Network database was performed. This is a prospectively maintained database covering four health boards (Ayrshire and Arran, Forth Valley, Greater Glasgow and Clyde and Lanarkshire) and includes approximately half the Scottish population. All patients diagnosed with colorectal cancer between January 2011 and December 2014 were identified in the registry database. Patients with rectal cancer, who presented as an emergency or who underwent surgery without curative intent, were excluded. This resulted in TNN I–III patients who underwent elective curative surgery in the cohort analysed. Following this, a subcohort of TNM III patients was identified for analysis. Emergency presentation was defined as an unplanned admission requiring urgent investigation and definitive management within 72 hours of presentation. No other patients were excluded.

### Data set and variables

Age, sex, and ethnicity were collected. SED was derived from the SIMD (version 2012)^18^. The generated SIMD score was divided into quintiles for interpretation, with SIMD 1 representing the most deprived quintile. SIMD was then compared to clinicopathological factors including staging, co-morbidity, and 3-year survival. TNM category was defined by the American Joint Committee on Cancer (AJCC) and was delineated via pathology from histopathology reports. Tumours were considered right sided if they were located from the caecum up to the splenic flexure; tumours were considered left sided if they were located from (and including) the splenic flexure to the rectosigmoid junction. Receipt of adjuvant chemotherapy was defined as receiving any form of chemotherapy after surgery regardless of the number of doses. Both the American Society of Anaesthesiology (ASA)^19^ grade and RCS Charlson Score^20^ were used to approximate co-morbidity. The RCS Charlson Score components were used to explore co-morbidity and its relationship with deprivation more deeply. Previous studies have shown that composite scores, such as the Systemic Inflammatory Grade (SIG)^21^, modified Glasgow Prognostic Score (mGPS)^21,22^, and C-reactive protein (CRP)/albumin ratio (CAR)^23^ alongside CRP, can quantify the systemic inflammatory response and have prognostic benefit in many different cohorts, including colon and rectal cancer. In the present study, CAR was selected for the multivariable analysis because it was the most sensitive of the composite scores for inflammation. Body mass index (BMI) was used to assess nutritional status. Extramural venous invasion (EVMI) positivity is associated with adverse prognosis and was included in the tumour factors^24^ . Most of the data were sourced from the Managed Clinical Network database, but data were also obtained from clinical records, including information on ethnicity, RCS Charlson Score, smoking status, BMI, SIG, mGPS, and CAR, alongside missing information. Data linkage from national death records was performed to obtain 30-day and 90-day mortality, as well as overall and cancer-specific survival data.

### Outcomes of interest

The primary outcome of interest was to examine the association between SED in patients undergoing surgery with curative intent for TNM I–III and TNM III colon cancer and both short-term (30-day and 90-day day mortality) and mid-term (3-year) survival outcomes. For outcomes, 30-day and 90-day mortality were defined as the time from surgery to death. Survival was also defined from the time of surgery to death after excluding deaths within 30 days of surgery. Overall survival (OS) was defined as death from any cause and cancer-specific survival (CSS) was defined as death directly resulting from the malignancy, as recorded on death certificates. The minimum follow-up time recorded for survival was at 3 years.

A secondary outcome of the study was to examine the association between adjuvant chemotherapy administration and SED in patients undergoing surgery with curative intent for colon cancer. Finally, associations between tumour and clinical factors and SED were tested (overall and in TNM III patients), and multivariable analysis was conducted to correlate these findings with survival.

### Ethics approval and consent to participate

Ethics approval for this study was obtained from the Public Beneficiary and Privacy Panel for Scotland. Due to the secondary pre-existing nature of the current study's data, ethics approval was granted in lieu of consent as per the Declaration of Helsinki.

### Statistical analysis

The associations between SIMD and primary short-term outcomes (30-day and 90-day mortality) were examined using χ^2^ tests. Primary mid-term survival outcomes; 3-year percentage OS and CSS were calculated overall and for each individual TNM stage using the lifetable function in SPSS^®^ version 25.0 (IBM, Armonk, NY, USA) with significance analysed using log-rank tests. Secondary outcomes, including associations of SIMD with adjuvant chemotherapy administration and tumour and host factors, were also examined using χ^2^ tests.

Then, factors with *P* < 0.100 were considered for multivariable regression analysis. Binary logistic regression analysis was used to assess factors related to SED with collation of SIMD categories to generate a deprived (SIMD1–2) and affluent (SIMD 4–5) group for comparison. Results are reported as odds ratios (ORs) and with their 95% confidence interval (c.i.). The association between significant clinicopathological factors (*P* < 0.100 on χ^2^ testing) and OS was examined using Cox regression, with results been reported as hazard ratios (HRs) and their 95% c.i. A subsequent Cox regression analysis was performed on CSS. Both for OS and CSS a backwards stepwise conditional approach was used. Patients with BMI <20 kg/m^2^ were excluded from the regression analysis because there were too few for meaningful analysis. SPSS^®^ version 25.0 (IBM, Armonk, NY, USA) was used for statistical analyses, with *P* < 0.05 considered statistically significant in regression analyses. Patients with missing variables were censored during the analysis.

## Results

### Study cohort

**Fig. 1:**
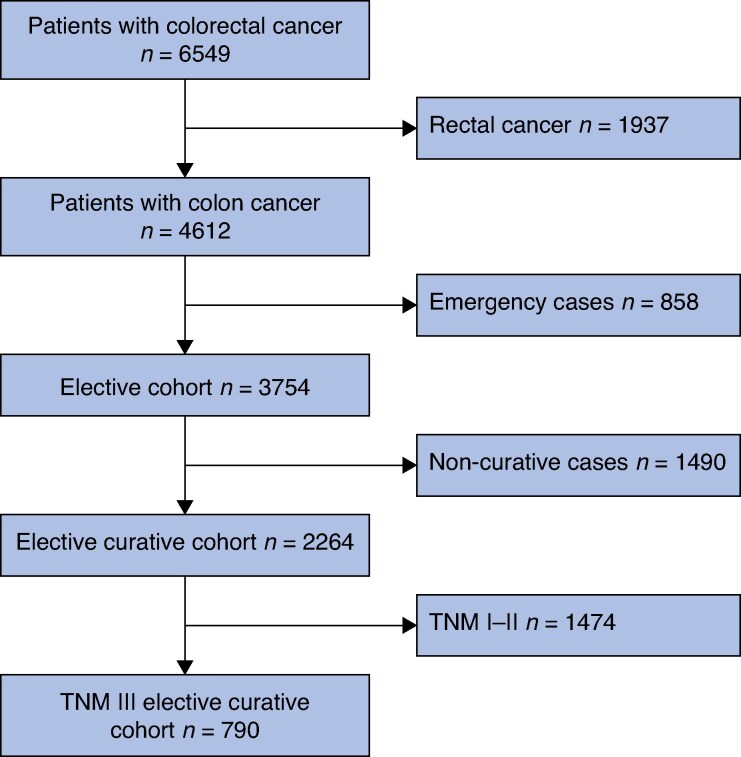
TNM I–III and III elective curative cancer cohorts .

During the study period (2011–2014), 6549 patients with colorectal cancer were identified in the West of Scotland Colorectal Cancer Managed Clinical Network database. Of these, 4612 patients (70.4%) had colon cancer, with 81.4% (3754) of patients with colon cancer presented electively. Of these patients, 2264 underwent surgery with curative intent. A further subgroup of TNM III elective curative cancer patients was delineated (790), as indicated in *Fig. 1*.

### Association between SED and short-term and mid-term survival

The median follow-up time was 72 months (range 43–95 months). The association between SIMD and short-term and mid-term outcomes is presented in *Table 1*. Overall, for TNM I–III patients considered as a single group, there was no association between SIMD and 30-day or 90-day postoperative mortality (*P* = 0.480 and *P* = 0.616, respectively). SED was associated with adverse 3-year OS (83 *versus* 86% for SIMD 1 and SIMD 5, respectively; *P* = 0.008) and CSS (90 *versus* 92% for SIMD 1 and SIMD 5, respectively; *P* = 0.024).

**Table 1:** Association between SIMD and mortality, survival, and receipt of adjuvant chemotherapy in elective curative colon cancer

	Total	SIMD 1	SIMD 2	SIMD 3	SIMD 4	SIMD 5	P*
**Postoperative mortality**							
TNM I–III	2264 (MN = 0)	599 (26.5%)	501 (22.1%)	406 (17.9%)	364 (16.1)	394 (17.4%)	
30-day mortality	43 (1.9%)	14 (2.3%)	5 (1.0%)	8 (2.0%)	9 (2.5%)	7 (1.8%)	0.480
90-day mortality	61 (2.7%)	19 (3.2%)	10 (2.0%)	13 (3.2%)	11 (3.0%)	8 (2.0%)	0.616
TNM III	790 (MN = 0)	217 (27.5%)	172 (21.8%)	147 (18.6%)	113(14.3%)	141 (17.8%)	
30-day mortality	22 (2.8%)	10 (4.6%)	3 (1.7%)	2 (1.4%)	4 (3.5%)	3 (2.1%)	0.296
90-day mortality	33 (4.1%)	13 (6.0%)	8 (4.7%)	5 (3.4%)	4 (3.5%)	3 (2.1%)	0.449
**Receipt of adjuvant chemotherapy**							
TNM I–III	2259 (MN = 5)	599 (26.5%)	501 (22.2%)	405 (17.9%)	362 (16.0%)	392 (17.4%)	0.152
No	1574 (69.7%)	423 (70.6%)	361 (72.0%)	274 (67.7%)	260 (71.8%)	256 (65.3%)	
Yes	685 (30.3%)	176 (29.4%)	140 (27.9%)	131 (32.3%)	102 (28.2%)	136 (34.7%)	
TNM III	788 (MN = 2)	217 (27.5%)	172 (21.8%)	146 (18.5%)	112 (14.2%)	141 (17.9%)	0.059
No	292 (37.1%)	88 (40.6%)	72 (41.9%)	54 (37.0%)	40 (35.7%)	38 (27.0%)	
Yes	496 (62.9%)	129 (59.4%)	100 (58.1%)	92 (63.0%)	72 (64.2%)	103 (73.0%)	
**3-year survival**							
TNM I–III	2221 (MN = 43)	585 (26.3%)	496 (22.3%)	398 (17.9%)	355 (16.0%)	387 (17.4%)	
CSS (%)	91 (SE1%)	90 (SE1%)	89 (SE1%)	90 (SE2%)	93 (SE1%)	92 (SE1%)	0.024
OS (%)	85 (SE1%)	83 (SE2%)	83 (SE2%)	84 (SE2%)	90 (SE2%)	86 (SE2%)	0.008
TNM III	768 (MN = 22)	207 (27%)	169 (22%)	145 (19%)	109 (14%)	138 (18%)	
CSS (%)	81 (SE1%)	78 (SE3%)	78 (SE3%)	81 (SE3%)	89 (SE3%)	82 (SE3%)	0.082
OS (%)	76 (SE2%)	72 (SE3%)	72 (SE3%)	75 (SE4%)	87 (SE3%)	76 (SE4%)	0.024

Values are *n* (%) unless otherwise stated. *P* values were generated though χ^2^ and Log rank testing. SIMD, Scottish Index of Multiple Deprivation; TNM, tumour node metastasis staging system; CSS, cancer-specific survival; OS, overall survival; MN, missing number; SE, standard error.

For TNM III disease, there was no association between SIMD and 30-day or 90-day postoperative mortality (*P* = 0.296 and *P* = 0.449, respectively). SED was associated with adverse 3-year OS (72 *versus* 76% for SIMD 1 and SIMD 5, respectively; *P* = 0.024). A similar trend association was seen for CSS, but failed to reach statistical significance (78 *versus* 82% for SIMD 1 and SIMD 5, respectively; *P* = 0.082; *Table 1*).

### Association between adjuvant chemotherapy administration and SED

The association between SIMD and the receipt of adjuvant chemotherapy is also presented in *Table 1*. Overall, for TNM I–III patients, there was no statistically significant association between SIMD and the receipt of adjuvant chemotherapy, 29.4% of SIMD 1 patients and 34.7% of SIMD 5 patients receiving adjuvant chemotherapy (*P* = 0.152). For the TNM III cohort, there was a trend between higher SED and a lower likelihood or receiving adjuvant chemotherapy, with 59.4% of SIMD 1 patients and 73.0% of SIMD 5 patients receiving adjuvant chemotherapy, although statistical significance was not reached (*P* = 0.059).

### Association between SIMD and clinicopathological features in the TNM I–III cohort

The association between SIMD and clinicopathological variables in 2264 patients undergoing elective curative resection surgery for TNM stage I–III colon cancer is presented in *Table 2*. A higher proportion of SIMD 1 than SIMD 5 patients had ASA grade III (36.6 *versus* 22.2%; *P* < 0.001) and IV (4.6 *versus* 1.8%; *P* < 0.001). Similarly, a higher proportion of SIMD 1 than SIMD 5 patients had a RCS Charlson Score of 2 (16.4 *versus* 11.5%; *P* < 0.001), were ex-smokers (43.8 *versus* 40.7%; *P* < 0.001) and current smokers (17.5 *versus* 4.0%; *P* < 0.001), had a BMI ≥ 30 kg/m^2^ (36.0 *versus* 22.7%; *P* < 0.001), had a mGPS of 2 (18.5 *versus* 14.2%; *P* = 0.007), and CRP ≥ 10 mg/L (35.6 *versus* 27.4%; *P* < 0.001) or CAR >0.2 (42.5 *versus* 31.7%; *P* = 0.003), although there was no overall trend across quintiles. There was no significant difference between SIMD groups for age (*P* = 0.924), sex (*P* = 0.386), ethnicity (*P* = 0.954), or SIG (*P* = 0.855).

**Table 2:** Association between SIMD and tumour/host factors in TNM I–III elective curative colon cancer

	Total	SIMD 1	SIMD 2	SIMD 3	SIMD 4	SIMD 5	P*
**Age (years)**	2264 (MN = 0)	599 (26.5%)	501 (22.1%)	406 (17.9%)	364 (16.1)	394 (17.4%)	0.924
< 65	670 (29.6%)	188 (31.4%)	144 (28.7%)	111 (27.3%)	113 (31.0%)	114 (28.9%)	
65–74	841 (37.1%)	222 (37.0%)	190 (37.9%)	151 (37.1%)	131 (35.9%)	147 (37.3%)	
≥ 75	753 (33.3%)	189 (31.5%)	167 (33.3%)	144 (35.5%)	120 (32.9%)	133 (33.7%)	
**Sex**	2264 (MN = 0)	599 (26.5%)	501 (22.1%)	406 (17.9%)	364 (16.1)	394 (17.4%)	0.386
Male	1204 (53.1%)	314 (52.4%)	252 (50.2%)	231 (56.9%)	196 (54%)	211 (53.8%)	
Female	1060 (46.8%)	285 (47.5%)	249 (49.7%)	175 (43.1%)	168 (46%)	183 (46.1%)	
**Ethnicity**	1396 (MN = 868)	402 (28.8%)	226 (16.2%)	203 (14.5%)	207 (14.8%)	258 (18.5%)	0.954
White British	1380 (98.9%)	396 (98.5%)	224 (99.1%)	200 (98.5%)	205 (99%)	255 (98.8%)	
Other	16 (1.1%)	6 (1.5%)	2 (0.9%)	3 (1.5%)	2 (1.0%)	3 (1.2%)	
**ASA grade**	2180 (MN = 84)	585 (26.8%)	482 (22.1%)	389 (17.8%)	345 (15.8%)	379 (17.4%)	< 0.001
I	224 (10.3%)	33 (5.6%)	50 (10.4%)	39 (10.0%)	45 (13.0%)	57 (15.0%)	
II	1247 (57.2%)	317 (54.2%)	270 (56.0%)	208 (53.5%)	221 (64.1%)	231 (60.9%)	
III	646 (29.6%)	208 (36.6%)	146 (30.3%)	134 (34.4%)	74 (21.4%)	84 (22.2%)	
IV	63 (2.9%)	27 (4.6%)	16 (3.3%)	8 (2.1%)	5 (1.4%)	7 (1.8%)	
**RCS Charlson Score**	2236 (MN = 28)	592 (26.5%)	494 (22.1%)	400 (17.9%)	359 (16.1%)	391 (17.5%)	< 0.001
0	1122 (50.1%)	252 (42.6%)	243 (49.2%)	219 (54.8%)	192 (53.5%)	216 (55.2%)	
1	678 (30.3%)	204 (34.5%)	142 (28.7%)	111 (27.8%)	108 (30.1%)	113 (28.9%)	
2	339 (15.2%)	97 (16.4%)	90 (18.2%)	54 (13.5%)	53 (14.8%)	45 (11.5%)	
≥ 3	97 (4.3%)	39 (6.6%)	19 (3.8%)	16 (4.0%)	6 (1.7%)	17 (4.3%)	
**Smoking status**	2152 (MN = 112)	582 (27.0%)	472 (21.9%)	384 (17.8%)	336 (15.6%)	378 (17.6%)	< 0.001
Non-smoker	1026 (47.7%)	225 (38.7%)	220 (46.6%)	186 (48.4%)	186 (55.4%)	209 (55.3%)	
Ex-smoker	875 (40.7%)	255 (43.8%)	189 (40.0%)	152 (49.6%)	125 (37.2%)	154 (40.7%)	
Smoker	251 (11.7%)	102 (17.5%)	63 (13.3%)	46 (12.0%)	25 (7.4%)	15 (4.0%)	
**BMI (kg/m^2^)**	1634 (MN = 630)	461 (28.2%)	361 (22.1%)	270 (16.5%)	247 (15.6%)	295 (17.6%)	< 0.001
< 20	79 (4.8%)	17 (3.7%)	28 (7.8%)	12 (4.4%)	5 (2.0%)	17 (5.8%)	
20–24.9	463 (28.3%)	119 (25.8%)	93 (25.8%)	79 (29.3%)	63 (25.5%)	109 (37.0%)	
25–29.9	577 (35.3%)	159 (34.5%)	118 (32.7%)	89 (33.0%)	109 (44.1%)	102 (34.6%)	
≥ 30	515 (31.5%)	166 (36.0%)	122 (33.8%)	90 (33.3%)	70 (28.3%)	67 (22.7%)	
**SIG**	1300 (MN = 964)	354 (27.2%)	296 (22.8%)	234 (18.0%)	200 (15.4%)	216 (16.6%)	0.855
0	532 (40.9%)	149 (4.1%)	116 (39.2%)	93 (39.7%)	77 (38.5%)	97 (45.0%)	
1	347 (26.7%)	87 (24.6%)	80 (27.0%)	69 (29.5%)	52 (26.0%)	59 (27.3%)	
2	237 (18.2%)	68 (19.2%)	53 (17.9%)	41 (17.5%)	46 (23.0%)	29 (13.4%)	
3	101 (7.8%)	26 (7.3%)	27 (9.1%)	17 (7.3%)	15 (7.5%)	16 (7.4%)	
4	83 (6.4%)	24 (6.8%)	20 (6.8%)	14 (6.0%)	10 (5.0%)	15 (6.9%)	
**mGPS**	1310 (MN = 954)	357 (27.3%)	297 (22.7%)	236 (18.0%)	202 (15.4%)	218 (16.6%)	0.007
0	888 (67.8%)	240 (67.2%)	184 (62.0%)	167 (70.8%)	133 (66.8%)	164 (75.2%)	
1	212 (16.2%)	51 (14.3%)	69 (23.2%)	36 (15.3%)	33 (16.3%)	23 (10.6%)	
2	210 (16.0%)	66 (18.5%)	44 (14.8%)	33 (14.0%)	36 (17.8%)	31 (14.2%)	
**C-Reactive protein**	1351 (MN = 913)	365 (27.0%)	312 (23.1%)	244 (18.1%)	207 (15.3%)	223 (16.5%)	< 0.001
< 3	255 (18.9%)	70 (19.2%)	32 (10.3%)	36 (14.8%)	37 (17.9%)	80 (35.9%)	
3–10	639 (47.3%)	165 (45.2%)	158 (50.6%)	134 (54.9%)	100 (48.3%)	82 (36.8%)	
≥ 10	457 (33.8%)	130 (35.6%)	122 (39.1%)	74 (30.3%)	70 (33.8%)	61 (27.4%)	
**CAR**	1311 (953)	358 (27.3%)	297 (22.7%)	236 (18.0%)	202 (15.4%)	218 (16.6%)	0.003
< 0.2	799 (61.0%)	206 (57.5%)	157 (52.9%)	154 (65.3%)	122 (60.4%)	149 (68.3%)	
> 0.2	523 (39.9%)	152 (42.5%)	140 (47.1%)	82 (34.7%)	80 (39.6%)	69 (31.7%)	
**TNM**	2264 (MN = 0)	599 (26.5%)	501 (22.1%)	406 (17.9%)	364 (16.1%)	394 (17.4%)	0.303
I	539 (23.8%)	137 (22.9%)	127 (25.3%)	79 (19.5%)	100 (27.5%)	96 (24.4%)	
II	935 (41.3%)	245 (40.9%)	202 (40.3%)	180 (44.3%)	151 (41.5%)	157 (39.9%)	
III	790 (34.9%)	217 (36.2%)	172 (34.3%)	147 (36.2%)	113 (31.0%)	141 (35.8%)	
**T category**	2260 (MN = 4)	599 (26.5%)	500 (22.1%)	404 (17.9%)	363 (16.1%)	394 (17.4%)	0.021
1	283 (12.5%)	82 (13.7%)	71 (14.2%)	37 (9.2%)	55 (15.2%)	38 (9.6%)	
2	346 (15.3%)	78 (13.0%)	72 (14.4%)	57 (14.1%)	60 (16.5%)	79 (20.1%)	
3	1158 (51.2%)	319 (53.3%)	243 (48.6%)	224 (55.4%)	172 (47.4%)	200 (50.8%)	
4	473 (20.9%)	120 (20.0%)	114 (22.8%)	86 (21.3%)	76 (20.9%)	77 (19.5%)	
**N category**	2264 (MN = 0)	599 (26.5%)	501 (22.1%)	406 (17.9%)	364 (16.1%)	394 (17.4%)	0.778
0	1474 (65.1%)	382 (63.8%)	329 (65.7%)	259 (63.8%)	251 (69.0%)	253 (64.2%)	
1	522 (23.1%)	142 (23.7%)	112 (22.4%)	103 (25.4%)	72 (19.8%)	93 (23.6%)	
2	268 (11.8%)	75 (12.5%)	60 (12.0%)	44 (10.8%)	41 (11.2%)	48 (12.1%)	
**Tumour side**	2246 (MN = 18)	594 (26.4%)	498 (22.2%)	401 (17.9%)	363 (16.2%)	390 (17.4%)	0.426
Right	1196 (53.3%)	327 (55.1%)	256 (51.4%)	225 (56.1%)	190 (52.3%)	198 (50.8%)	
Left	1050 (46.7%)	267 (44.9%)	242 (48.6%)	176 (43.9%)	173 (47.7%)	192 (49.2%)	
**Differentiation**	2254 (MN = 10)	597 (26.5%)	500 (22.2%)	403 (17.9%)	362 (16.1%)	392 (17.4%)	0.707
Moderate–well	1875 (83.2%)	503 (84.3%)	416 (83.2%)	326 (80.9%)	301 (83.1%)	329 (83.9%)	
Poor	379 (16.8%)	94 (15.7%)	84 (16.8%)	77 (19.1%)	61 (16.9%)	63 (16.1%)	
**EVMI**	2218 (MN = 46)	587 (26.5%)	492 (22.2%)	397 (17.9%)	353 (15.9%)	389 (17.5%)	0.090
Negative	1375 (62.0%)	352 (60.0%)	317 (64.4%)	232 (58.4%)	236 (66.9%)	238 (61.2%)	
Positive	843 (38.0%)	235 (40.0%)	175 (35.6%)	165 (41.6%)	117 (33.1%)	151 (38.8%)	

Values are *n* (%) unless otherwise stated. *P* values were generated using χ^2^. SIMD, Scottish Index of Multiple Deprivation; TNM, tumour node metastasis staging system; ASA, American Society of Anesthesiologists; BMI, body mass index; SIG, systemic inflammatory grade; mGPS, Modified Glasgow Prognostic Score; CAR, C-reactive protein to albumin ratio; EVMI, extramural venous invasion; MN, missing number.

There was an association between SIMD 1 *versus* SIMD 5 and more advanced T category (20.5 *versus* 19.5%, respectively, for T4; *P* = 0.021). No significant association was seen between SIMD and TNM category (*P* = 0.303), N category (*P* = 0.778), differentiation (*P* = 0.707), or EVMI positivity (*P* = 0.090).

The association between SIMD and individual co-morbidity factors is presented in *Table S1*. For TNM I–III, when individual co-morbidity factors were examined, SIMD 1 *versus* SIMD 5 patients had a higher proportion of lung disease (17.2 *versus* 12.5%; *P* = 0.020), previous myocardial infarction (9.0 *versus* 4.1%; *P* = 0.007), and peripheral vascular disease (4.2 *versus* 2.0%; *P* = 0.012). There was a trend between higher SED and diabetes, but this did not reach statistical significance (*P* = 0.061). No differences were found across SIMD groups for congestive heart failure, cerebrovascular accident, dementia, liver disease, renal disease, or rheumatoid disease (all *P* > 0.05). No patients in the cohort had HIV.

Results from multivariable analyses of associations between SIMD and tumour or host factors are presented in *Table 3*. ASA grade (OR 1.70; 95% c.i. 1.31 to 2.20; *P* < 0.001), smoking status (OR 1.59, 95% c.i. 1.24 to 2.03; *P* < 0.001), and BMI (OR 1.23; 95% c.i. 1.01 to 1.50; *P* = 0.045) were shown to be independently associated with SIMD 1–2. A separate analysis with RCS Charlson Score rather than ASA grade showed that the RCS Charlson Score was associated with SIMD 1–2 (OR 1.22; 95% c.i. 1.01 to 1.47; *P* = 0.039), but because the ASA grade was more strongly associated with deprivation, with greater statistical significance, it was preferentially used in the multivariable analysis for survival.

**Table 3:** Association between SIMD and clinicopathological factors for TNM I–III and TNM III: multivariable analysis

	ASA		RCS Charlson Score	
	OR	*P*	OR	*P*
**TNM I–III**				
ASA grade	1.70 (1.31, 2.20)	< 0.001		
Charlson Index			1.22 (1.01, 1.47)	0.039
Smoking	1.59 (1.24, 2.03)	< 0.001	1.61 (1.27, 2.05)	< 0.001
BMI	1.23 (1.01, 1.50)	0.045	1.23 (1.01, 1.50)	0.041
CAR		0.426		0.361
Diabetes		0.425		0.283
Lung disease		0.257		0.219
MI		0.583		0.368
PVD		0.791		0.569
T category		0.681		0.770
EVMI		0.573		0.574
**TNM stage III**				
ASA grade	1.58 (1.08, 2.31)	0.020		
MI		0.847		
PVD		0.236		
Smoking	1.52 (1.00, 2.32)	0.051		
BMI		0.111		
Receipt of AC		0.798		
CAR		0.423		

Values in parentheses are 95% confidence intervals. SIMD, Scottish Index of Multiple Deprivation; TNM, tumour node metastasis staging system; ASA, American Society of Anesthesiologists; OR, odds ratio; BMI, body mass index; CAR, C-reactive protein to albumin ratio; MI, myocardial infarction; PVD, peripheral vascular disease; EVMI, extramural venous invasion; AC, adjuvant chemotherapy.

### Associations between SIMD and clinicopathological features in TNM III patients

The associations between SIMD and tumour and host factors in patients undergoing elective curative resectional surgery for TNM III colon cancer are presented in *Table 4* A higher proportion of SIMD 1 than SIMD 5 patients had ASA grade III (39.3 *versus* 21.4%; *P* < 0.001) and IV (4.3 *versus* 1.5%; *P* < 0.001). Similarly, a higher proportion of SIMD 1 than SIMD 5 patients were ex-smokers (46.7 *versus* 44.5%; *P* = 0.004) and current smokers (11.9 *versus* 4.4%; *P* = 0.004), had a BMI ≥ 30 kg/m^2^ (37.9 *versus* 24.2%; *P* = 0.030), CRP ≥10 mg/L (37.6 *versus* 34.5%; *P* = 0.007), or CAR >0.2 (46.8 *versus* 37.8%; *P* < 0.001), although there was no overall trend across quintiles. There was no significant difference between SIMD groups for age (*P* = 0.420), sex (*P* = 0.417), ethnicity (*P* = 0.407), RCS Charlson Score (*P* = 0.217), SIG (*P* = 0.326), or mGPS (*P* = 0.146).

**Table 4:** Association between SIMD and tumour/host factors in TNM III elective curative colon cancer

	Total	SIMD 1	SIMD 2	SIMD 3	SIMD 4	SIMD 5	P*
**Age (years)**	790 (MN = 0)	217 (27.5%)	172 (21.8%)	147 (18.6%)	113 (14.3%)	141 (17.8%)	0.420
< 65	256 (32.4%)	82 (37.8%)	43 (25.0%)	47 (32.0%)	39 (33.5%)	45 (31.9%)	
65–74	264 (33.4%)	64 (29.5%)	62 (36.0%)	50 (34.0%)	38 (33.6%)	50 (35.4%)	
≥ 75	270 (34.2%)	71 (32.7%)	67 (39.0%)	50 (34.0%)	36 (31.9%)	46 (32.6%)	
**Sex**	790 (MN = 0)	217 (27.5%)	172 (21.8%)	147 (18.6%)	113 (14.3%)	141 (17.8%)	0.417
Male	409 (51.8%)	118 (54.4%)	78 (45.3%)	77 (52.4%)	62 (54.9%)	74 (52.5%)	
Female	381 (48.2%)	99 (45.6%)	94 (54.7%)	70 (47.6%)	51 (45.1%)	67 (47.5%)	
**Ethnicity**	458 (MN = 332)	147 (32.1%)	78 (17.0%)	72 (15.7%)	71 (15.5%)	90 (19.7%)	0.407
White British	447 (97.6%)	141 (95.9%)	77 (98.7%)	70 (97.2%)	71 (100.0%)	88 (97.8%)	
Other	11 (2.4%)	6 (4.1%)	1 (1.3%)	2 (2.8%)	0 (0.0%)	2 (2.22%)	
**ASA grade**	753 (MN = 37)	211 (28.0%)	163 (21.6%)	140 (18.6%)	108 (14.3%)	131 (17.4%)	< 0.001
I	80 (10.6%)	15 (7.1%)	18 (11.0%)	10 (7.1%)	16 (14.8%)	21 (16.0%)	
II	423 (56.2%)	104 (59.3%)	91 (55.8%)	80 (57.1%)	68 (63.0%)	80 (61.1%)	
III	225 (39.9%)	83 (39.3%)	44 (27.0%)	49 (35.0%)	21 (19.4%)	28 (21.4%)	
IV	25 (3.3%)	9 (4.3%)	10 (6.1%)	1 (0.7%)	3 (2.8%)	2 (1.5%)	
**RCS Charlson Score**	779 (MN = 11)	215 (27.6%)	167 (21.4%)	144 (18.5%)	112 (14.4%)	141 (18.1%)	0.217
0	389 (50.0%)	91 (42.3%)	85 (50.9%)	78 (54.2%)	62 (55.4%)	73 (51.8%)	
1	231 (29.7%)	77 (35.8%)	40 (24.0%)	40 (27.8%)	33 (29.5%)	41 (29.1%)	
2	125 (16.0%)	34 (15.8%)	34 (20.4%)	22 (15.3%)	15 (13.4%)	20 (14.2%)	
≥ 3	34 (4.4%)	13 (6.0%)	8 (4.7%)	4 (2.8%)	2 (1.8%)	7 (5.0%)	
**Smoking status**	749 (MN = 41)	210 (28.0%)	156 (20.8%)	138 (18.4%)	108 (14.4%)	137 (18.3%)	0.004
Non-smoker	368 (49.1%)	87 (41.4%)	71 (45.5%)	70 (50.7%)	70 (64.8%)	70 (51.1%)	
Ex-smoker	314 (41.9%)	98 (46.7%)	71 (45.5%)	52 (37.7%)	32 (29.6%)	61 (44.5%)	
Smoker	67 (8.9%)	25 (11.9%)	14 (9.0%)	16 (11.6%)	6 (5.6%)	6 (4.4%)	
**Body mass index (kg/m^2^)**	650 (MN = 140)	177 (27.2%)	139 (21.4%)	112 (17.2%)	94 (14.5%)	128 (19.7%)	0.030
<20	37 (5.7%)	7 (4.0%)	14 (10.1%)	4 (3.6%)	3 (3.2%)	9 (7.0%)	
20–24.9	182 (28.0%)	48 (27.1%)	31 (22.3%)	32 (28.6%)	24 (25.5%)	47 (36.7%)	
25–29.9	222 (34.2%)	55 (31.1%)	45 (32.4%)	39 (38.4%)	42 (44.7%)	41 (32.0%)	
≥ 30	209 (32.2%)	67 (37.9%)	49 (35.3%)	37 (33.0%)	25 (26.6%)	31 (24.2%)	
**Systemic inflammatory grade**	473 (MN = 317)	137 (29.0%)	105 (22.2%)	85 (18.0%)	64 (13.5%)	82 (17.3%)	0.326
0	179 (37.8%)	52 (38.0%)	36 (34.3%)	34 (40.0%)	21 (32.8%)	36 (43.9%)	
1	127 (26.8%)	36 (26.3%)	24 (22.9%)	28 (32.9%)	18 (28.1%)	21 (25.6%)	
2	82 (17.3%)	28 (20.4%)	18 (17.1%)	15 (17.6%)	13 (20.3%)	8 (9.8%)	
3	47 (9.9%)	11 (8.0%)	17 (16.2%)	5 (5.9%)	7 (10.9%)	7 (8.5%)	
4	38 (8.0%)	10 (7.3%)	10 (9.5%)	3 (3.5%)	5 (7.8%)	10 (12.2%)	
**Modified Glasgow Prognostic Score**	476 (MN = 314)	139 (29.2%)	105 (22.1%)	85 (17.9%)	65 (13.7%)	82 (17.2%)	0.146
0	292 (61.3%)	88 (63.3%)	51 (48.6%)	60 (70.6%)	39 (60.0%)	54 (65.9%)	
1	96 (20.2%)	26 (18.7%)	30 (28.6%)	14 (16.5%)	13 (20.0%)	13 (15.9%)	
2	88 (18.5%)	25 (18.0%)	24 (22.9%)	11 (12.9%)	13 (20.0%)	15 (18.3%)	
**CRP (mg/L)**	492 (MN = 298)	141 (28.7%)	112 (22.8%)	87 (17.7%)	68 (13.8%)	84 (17.1%)	0.007
<3	94 (19.1%)	29 (20.6%)	12 (10.7%)	14 (16.1%)	13 (19.1%)	26 (31.0%)	
3–10	208 (42.3%)	59 (41.8%)	44 (39.3%)	47 (54.0%)	29 (42.6%)	29 (34.5%)	
≥10	190 (38.6%)	53 (37.6%)	56 (50.0%)	26 (29.9%)	26 (38.2%)	29 (34.5%)	
**CRP/albumin ratio**	476 (MN = 314)	139 (29.2%)	105 (22.1%)	85 (17.9%)	65 (13.8%)	82 (17.2%)	< 0.001
<0.2	257 (54.0%)	74 (53.2%)	39 (37.1%)	57 (67.1%)	36 (55.4%)	51 (62.2%)	
>0.2	219 (46.0%)	65 (46.8%)	66 (62.9%)	28 (32.9%)	29 (44.6%)	31 (37.8%)	
**T category**	789 (MN = 1)	216 (27.4%)	171 (21.7%)	147 (18.6%)	114 (14.4%)	141 (17.9%)	0.466
1	27 (3.4%)	7 (3.2%)	9 (5.3%)	3 (2.0%)	4 (3.5%)	4 (2.8%)	
2	68 (8.6%)	16 (7.4%)	8 (4.7%)	14 (9.5%)	13 (11.4%)	17 (12.1%)	
3	414 (52.5%)	121 (56.0%)	87 (50.9%)	75 (51.0%)	56 (49.1%)	75 (53.2%)	
4	280 (35.5%)	72 (33.3%)	67 (39.2%)	55 (37.4%)	41 (36.0%)	45 (31.9%)	
**N category**	789 (MN = 1)	216 (27.4%)	171 (21.7%)	147 (18.6%)	114 (14.4%)	141 (17.9%)	0.854
1	522 (66.2%)	141 (65.3%)	112 (65.5%)	103 (70.1%)	73 (64.0%)	93 (66.0%)	
2	267 (33.8%)	75 (34.7%)	59 (34.5%)	44 (30.0%)	41 (36.0%)	48 (34.0%)	
**Tumour side**	781 (MN = 9)	213 (27.3%)	170 (21.8%)	145 (18.6%)	114 (14.9%)	139 (18.6%)	0.850
Right	406 (52.0%)	114 (53.5%)	92 (54.1%)	75 (51.7%)	58 (50.9%)	67 (48.2%)	
Left	375 (48.0%)	99 (46.5%)	78 (45.9%)	70 (48.3%)	56 (49.1%)	72 (51.8%)	
**Differentiation**	787 (MN = 3)	216 (27.4%)	171 (21.7%)	146 (18.6%)	114 (14.5%)	140 (17.8%)	0.458
Moderate–well	604 (76.7%)	170 (78.7%)	124 (72.5%)	109 (74.7%)	92 (80.7%)	109 (77.9%)	
Poor	183 (23.3%)	46 (21.3%)	47 (27.5%)	37 (25.3%)	22 (19.3%)	31 (22.1%)	
**EVMI**	785 (MN = 5)	216 (27.5%)	168 (21.4%)	147 (18.7%)	113 (14.4%)	141 (18.0%)	0.904
Negative	316 (40.3%)	82 (38.0%)	68 (40.5%)	61 (41.5%)	49 (43.4%)	56 (39.7%)	
Positive	469 (59.7%)	134 (62.0%)	100 (59.5%)	86 (58.5%)	64 (56.6%)	85 (60.3%)	

Values are *n* (%) unless otherwise stated. *P* values were generated through χ^2^. SIMD, Scottish Index of Multiple Deprivation; TNM, tumour node metastasis staging system; ASA, American Society of Anesthesiologists; CRP, C-reactive protein; EVMI, extramural venous invasion; MN, missing number.

No association was seen between SIMD and tumour factors, including T category (*P* =0.466), N category (*P* = 0.854), tumour side (*P* = 0.850), tumour differentiation (*P*= 0.456), EVMI positivity (*P* = 0.904), and perineural invasion (*P* = 0.361).

Results from multivariable analyses of associations between SIMD and tumour or host factors in TNM III colon cancer are also presented in *Table 3*. ASA grade was independently associated with SIMD 1–2 (OR 1.58; 95% c.i. 1.08 to 2.31; *P* = 0.020). Although there was an association between SED and smoking (OR 1.52; 95% c.i. 1.00 to 2.32; *P* = 0.051), it failed to reach statistical significance.

For TNM III only disease, when comparing individual co-morbidity factors (*Table S1*), the SIMD 1 cohort had a higher proportion of previous myocardial infarction (10.2 *versus* 5.0%; *P* = 0.015) and peripheral vascular disease (4.3 *versus* 1.4%; *P* = 0.036) than the SIMD 5 cohort. No differences were found between SIMD groups for congestive heart failure, cerebrovascular accident, diabetes, dementia, liver disease, lung disease, renal disease, or rheumatoid disease (all *P* > 0.050). No patients in the cohort had HIV.

### Associations between SIMD, clinicopathological factors, and mid-term survival

Results from univariable and multivariable analyses between OSandtumour and host factors are presented in *Table 5*. On univariable analysis for TNM I–III, a statistically significant association was found between OS and SIMD (HR 0.93; 95% c.i. 0.86 to 0.99; *P* = 0.032), ASA grade (HR 1.64; 95% c.i. 1.40 to 1.91; *P* < 0.001), BMI (HR 0.85; 95% c.i. 0.75 to 0.97; *P* = 0.013), CAR (HR 1.56; 95% c.i. 1.05 to 1.28; *P* = 0.003), diabetes (HR 1.36; 95% c.i. 1.05 to 1.28; *P* = 0.020), previous myocardial infarction (HR 2.12; 95% c.i. 1.50 to 2.99; *P* < 0.001), peripheral vascular disease (HR 2.23; 95% c.i. 1.35 to 3.68; *P* = 0.002), T category (HR 2.03; 95% c.i. 1.76 to 2.34; *P* < 0.001), and EVMI (HR 2.01; 95% c.i. 1.63 to 2.47; *P* < 0.001). When the factors significant on univariable survival analysis were entered into a multivariable model, ASA grade (HR 1.57; 95% c.i. 1.27 to 1.95; *P* < 0.001), BMI (HR 0.82; 95% c.i. 0.69 to 0.98; *P* = 0.026), and T category (HR 1.69; 95% c.i. 1.41 to 2.03; *P* < 0.001) remained independently associated with OS.

**Table 5:** Association between clinicopathological factors and OS for TNM I–III and TNM III: univariable and multivariable analysis

	TNM I–III				TNM III			
	Univariable analysis		Multivariable analysis		Univariable analysis		Multivariable analysis	
	HR	*P*	HR	*P*	HR	*P*	HR	*P*
SIMD	0.93 (0.86, 0.99)	0.032		0.617	0.95 (0.86, 1.05)	0.029		0.960
ASA grade	1.64 (1.40, 1.91)	< 0.001	1.57 (1.27, 1.95)	< 0.001	1.57 (1.28, 1.93)	<0.001		0.086
Smoking		0.104				0.876		
BMI	0.85 (0.75, 0.97)	0.013	0.82 (0.69, 0.98)	0.026		0.451		
CRP/albumin ratio	1.56 (1.05, 1.28)	0.003		0.439	1.17 (1.06, 1.30)	0.002		0.067
Diabetes	1.36 (1.05, 1.76)	0.020		0.321				
Lung disease		0.431						
Myocardial infarction	2.12 (1.50, 2.99)	< 0.001		0.064	2.25 (1.37, 3.71)	< 0.001	1.97 (1.00, 3.86)	0.050
PVD	2.23 (1.35, 3.68)	0.002		0.271		0.860		
Receipt of AC					0.43 (0.32, 0.58)	< 0.001	0.39 (0.26, 0.58)	< 0.001
T category	2.03 (1.76, 2.34)	< 0.001	1.69 (1.41, 2.03)	< 0.001				
EVMI	2.01 (1.63, 2.47)	< 0.001		0.171				

Values in parentheses are 95% confidence intervals. TNM, tumour node metastasis staging system; HR, hazard ratio; SIMD, Scottish Index of Multiple Deprivation; ASA, American Society of Anesthesiologists; BMI, body mass index; CRP, C-reactive protein; PVD, peripheral vascular disease; AC, adjuvant chemotherapy; EVMI, extramural venous invasion.

On univariable analysis for TNM III disease, a statistically significant association was found between OS and SIMD (HR 0.95; 95% c.i. 0.86 to 1.05; *P* = 0.029), ASA grade (HR 1.57; 95% c.i. 1.28 to 1.93; *P* < 0.001), previous myocardial infarction (HR 2.25; 95% c.i. 1.37 to 3.71; *P* < 0.001), the receipt of adjuvant chemotherapy (HR 0.43; 95% c.i. 0.32 to 0.58; *P* < 0.001), and CAR (HR 1.17; 95% c.i. 1.06 to 1.30; *P* = 0.002). When the factors significant on univariable survival analysis were entered into a multivariable model, previous myocardial infarction (HR 1.97; 95% c.i. 1.00 to 3.86; *P* = 0.050) and the receipt of adjuvant chemotherapy (HR 0.39; 95% c.i. 0.26 to 0.58; *P* < 0.001) remained independently associated with OS.

The associations between CSS and tumour and host factors on multivariable analysis are presented in *Table S2*. On univariable analysis for TNM I–III, a statistically significant association was found between CCS and ASA grade (HR 1.26;, 95% c.i. 1.02 to 1.55; *P* = 0.032), CAR (HR 1.21; 95% c.i. 1.08 to 1.35; *P* < 0.001), T category (HR 3.41; 95% c.i. 2.74 to 4.25; *P* < 0.001), and EVMI (HR 3.31; 95% c.i. 2.48 to 4.42; *P* < 0.001). When the factors significant on univariable survival analysis were entered into a multivariable model, T category (HR 2.47; 95% c.i. 1.82 to 3.34; *P* < 0.001) and EMVI (HR 1.90; 95% c.i. 1.27 to 2.84; *P* = 0.002) remained independently associated with CSS.

On univariable analysis for TNM III disease, a statistically significant association was found between CSS and ASA grade (HR 1.30; 95% c.i. 1.02 to 1.65; *P* = 0.034), previous myocardial infarction (HR 1.88; 95% c.i. 1.01 to 3.47; *P* = 0.045), the receipt of adjuvant chemotherapy (HR 0.60; 95% c.i. 0.42 to 0.85; *P* = 0.004), and CAR (HR 1.24; 95% c.i. 1.08 to 1.43; *P* = 0.003). When the factors significant on univariable survival analysis were entered into a multivariable model, previous myocardial infarction (HR 2.19; 95% c.i. 1.00 to 4.78; *P* = 0.049), the receipt of adjuvant chemotherapy (HR 0.46; 95% c.i. 0.29 to 0.73; *P* < 0.001), and CAR (HR 1.24; 95% c.i. 1.07 to 1.44; *P* = 0.004) remained independently associated with CSS.

## Discussion

The results of the present study show that, on unadjusted analysis, SED was associated with adverse 3-year OS and CSS in patients undergoing resectional surgery with curative intent for colon cancer.

However, after adjustment for other tumour and host factors, SED was not independently associated with survival. In addition, SED was related to adverse host, but not tumour, characteristics. It therefore seems likely that the negative prognostic impact of SED is due, in part, to the impact of SED on host factors (including co-morbidity), and this association is responsible for the negative impact of SED on long-term OS.

The effect of SED in prescreening cohorts of colorectal cancer has previously been examined by Hole and McArdle^3^ and Oliphant *et al*.^2^. In keeping with the present unadjusted results, these authors reported that SED was associated with poorer survival. Other studies have also reported the negative impact of deprivation on survival^25,26^. Therefore, despite advances in the treatment of colon cancer across the past 20 years, including minimally invasive surgery, centralization of services, and the introduction of colorectal screening, socioeconomic inequality persists for these patients. The need to further explore why socioeconomically deprived patients have adverse long-term survival compared with their affluent counterparts is clear. Indeed, this has recently been acknowledged in the Scottish Government Cancer Strategy 2023–2033^27^.

The present study explored tumour factors to delineate the contributors behind this poor survival. There is little literature exploring tumour factors and SED except for stage at presentation. For stage, previous literature paints a mixed picture, with some studies demonstrating no association between stage at presentation and SED^2,26^ and others reporting that higher deprivation is associated with a more advanced stage at presentation^28,29^. In the present study, there was no clear association between SED and any included tumour factor; therefore, it is unlikely that SED results in a biologically different disease. It also explains the lack of association between SED and adverse CSS.

Co-morbidity has previously been linked to deprived populations^30^. In the present study, co-morbidity was associated with deprivation and was associated with adverse survival. The present study analysed co-morbidity both subjectively (ASA grade) and objectively (RCS Charlson Score). ASA grade in particular demonstrated a strong association with deprivation. ASA grade, although subjective, also captures an element of the patient’s condition that may represent a patient’s frailty or co-morbidity not captured by the RCS Charlson Score. This could explain why the RCS Charlson Score is not as strongly associated with deprivation. ASA grade has previously^31–33^ been linked to poor complication profiles and survival in elective patients. Further research into the links between SED and co-morbidity would require exploration of frailty/functional status and other co-morbidities not considered by the RCS Charlson Score.

Lifestyle factors both comprise a significant proportion of the health component of SED and are predictors of co-morbidity. Smoking and increased BMI are characteristic of deprived populations and are associated with an increased risk of colon cancer^15,34^. The present study identified an independent association between SIMD and both smoking and BMI on multivariable analysis. More recently, there has been increased interest in the stratification of muscle/fat mass through the use of routinely performed staging computed tomography scans, and this has been shown to be prognostic^35–37^. Further investigation of computed tomography-derived body composition within the context of SED and outcomes would be of interest.

In addition, lifestyle factors have been previously shown to be linked to higher cardiovascular co-morbidity and survival, with deprived populations being disproportionately affected^38^. With co-morbidity, the poor survival outcomes could be reduced by interventions such as prehabilitation, especially because there is evidence of some benefit in non-SED delineated cohorts^39^. Further trials are currently planned to explore the benefits of exercise and nutrition optimization for TNM III patients via the ENICTO trial^13^.

The present study found an association between the systemic inflammatory response on univariable but not multivariable analysis. An elevated systemic inflammatory response has previously been associated with smoking, increased BMI, SED, and outcomes^40–44^. With this mixed picture, further in-depth examination of inflammation in deprived populations is warranted.

Frailty has been associated with SED and adverse outcome in patients with colorectal cancer^45^. Although there is a strong association between age and frailty, younger patients can be considered frail. Frailty is typically considered a combination of co-morbidity, physical ability, and age. Deprived populations are more likely to smoke and have other co-morbidities, and therefore be exposed to inflammatory insults and so more likely to be considered frail despite their age. Therefore, not only is further in-depth assessment of inflammation required, but age and frailty must also be considered in the context of deprived populations.

The present study took place in the UK, which has a universal free-at-the-point-of-access health service. As a result, financial barriers to accessing healthcare are reduced, and this could explain the lack of statistically significant association of the receipt of chemotherapy with deprivation. This could also explain the lack of association of SIMD and stage at presentation, 30-day and 90-day postoperative mortality, and CSS. Nur *et al*.^46^ reported a similar lack of association with the receipt of adjuvant chemoradiotherapy in a UK-based trial. Despite the lack of statistical significance, there was an overall trend for TNM III patients with greater deprivation being less likely to receive chemotherapy; therefore, this is another area that requires further exploration.

The present study does have several limitations. SED has been linked to ethnicity^47^ and the present study population lacks diversity to explore this relationship further. In addition, the receipt of any adjuvant chemotherapy was explored in isolation and other factors, such as different types and duration of chemotherapy, were not considered. Due to the retrospective nature of data collection for composite variables (for example, systemic inflammatory grade and BMI) the present study suffers from missing data. The interrelation of the multiple lifestyle factors and co-morbidity raises a key issue in SED research. It is widely understood what SED is, but the multifactorial nature and variable scoring between populations generates difficulty in delineating causative relationships, requiring granularity of population data to overcome this. This may also mean the results reported here differ from those of other countries as a result. In addition, the study population in the present study has open access to a free-at-the-point-of-access healthcare service, which makes comparison to populations with different healthcare models difficult.

In conclusion, the results of the present study show that the adverse impact of SED on prognosis in patients undergoing resection surgery with curative intent for colon cancer is related to adverse host factors, as opposed to tumour biology. This may be a direct effect of co-morbidity on perioperative morbidity or all-cause mortality, or the lower life expectancy seen in individuals with increased SED. Alternatively, it may reflect the impact of SED and related co-morbidity on the use, or tolerance, of adjuvant therapy. Further investigation into the impact of SED on the host, including frailty, the systemic inflammatory response, and adjuvant chemotherapy, is warranted. It remains of interest whether prehabilitation including lifestyle modifications can improve outcomes in this high-risk group of patients.

## Supplementary Material

zrag014_Supplementary_Data

## Data Availability

This study brought together existing research data obtained from the West of Scotland Colorectal Cancer Managed Clinical Network Database and is available upon request from the Authors.
