# Supplementary material for: Impact of socioeconomic deprivation in patients undergoing elective surgical resection for colon cancer
Source: BJS Open. 2026 Mar 23;10(2):zrag014. doi: 10.1093/bjsopen/zrag014 (PMC13008328; doi:10.1093/bjsopen/zrag014)
Supplement: zrag014_Supplementary_Data [file zrag014_supplementary_data.docx]

**Impact of socioeconomic deprivation in patients undergoing elective surgical resection for colon cancer**

Sophie. M Tait^1,2^, Lucia Chung^3^, Paul. G. Horgan^1,2^, Campbell. S. D. Roxburgh^1,2^, Donald. C McMillan^1,2^ Allan. M Golder^1,2^.

Affiliations

1. Academic Unit of Surgery, Glasgow Royal Infirmary, Scotland, United Kingdom
2. University of Glasgow, University Avenue, Glasgow, G128QQ, Scotland, United Kingdom
3. Inverclyde royal hospital, Larkfield Road, Greenock, PA160XN

**Corresponding author.** Sophie M. Tait. [sophie.tait@glasgow.ac.uk](mailto:sophie.tait@glasgow.ac.uk) Orchid No: 0000-0002-1090-8624

**Supplementary Materials - Index**

| **Supplementary Figures and Tables** |  |
| --- | --- |
| Supplementary Table 1-Association between SIMD and comorbidity in elective presentations of TNM I-III and TNM stage III elective curative colon cancer | *page 3-4* |
| Supplementary Table 2-Association between clinicopathological factors and CSS for TNM I-III and TNM stage III-univariate and multivariate analysis (CSS). | *page 5* |

**Supplementary Figures and Tables**

Supplementary Table 1- Association between SIMD and comorbidity in elective presentations of TNM I-III and TNM stage III elective curative colon cancer.

| Comorbidity | Total | SIMD 1 | SIMD 2 | SIMD 3 | SIMD 4 | SIMD 5 | p |
| --- | --- | --- | --- | --- | --- | --- | --- |
| Congestive cardiac failure | | | | | | | |
| TNM I-III | 2236 | 592(26.5%) | 494(22.1%) | 400(17.9%) | 359(16.1%) | 391(17.5%) | 0.597 |
| No | 2171(97.1%) | 571(96.5%) | 479(97.0%) | 391(97.8%) | 347(96.7%) | 383(98.0%) |  |
| Yes | 65(2.9%) | 21(3.5%) | 15(3.0%) | 9(2.3%) | 12(3.3%) | 8(2.0%) |  |
| TNM III | 779 | 215(27.6%) | 167(21.4%) | 144(18.5%) | 112(14.4%) | 141(18.1%) | 0.832 |
| No | 753(96.7%) | 207(96.3%) | 160(95.8%) | 140(97.2%) | 110(98.2%) | 136(96.5%) |  |
| Yes | 26(3.3%) | 8(3.7%) | 7(4.2%) | 4(2.8%) | 2(1.8%) | 5(3.5%) |  |
| Cerebrovascular accident (CVA) | | | | | | | |
| TNM I-III | 2236 | 592(26.5%) | 494(22.1%) | 400(17.9%) | 359(16.1%) | 391(17.5%) | 0.371 |
| No | 2050(91.7%) | 539(91.0%) | 451(91.3%) | 376(94.0%) | 324(90.3%) | 360(92.0%) |  |
| Yes | 186(8.3%) | 53(9.0%) | 43(8.7%) | 24(6.0%) | 35(9.7%) | 31(7.9%) |  |
| TNM III | 779 | 215(27.6%) | 167(21.4%) | 144(18.5%) | 112(14.4%) | 141(18.1%) | 0.481 |
| No | 721(92.6%) | 198(92.1%) | 155(92.8%) | 138(95.8%) | 101(90.2%) | 129(91.5%) |  |
| Yes | 58(7.4%) | 17(7.9%) | 12(7.2%) | 6(4.2%) | 11(9.8%) | 12(8.5%) |  |
| Dementia | | | | | | | |
| TNM I-III | 2236 | 592(26.5%) | 494(22.1%) | 400(17.9%) | 359(16.1%) | 391(17.5%) | 0.386 |
| No | 2217(99.2%) | 584(98.6%) | 491(99.4%) | 399(99.8%) | 355(98.9%) | 388(99.2%) |  |
| Yes | 19(0.8%) | 8(1.4%) | 3(0.6%) | 1(0.3%) | 4(1.1%) | 3(0.8%) |  |
| TNM III | 779 | 215(27.6%) | 167(21.4%) | 144(18.5%) | 112(14.4%) | 141(18.1%) | 0.203 |
| No | 774(99.4%) | 215(100.0%) | 166(99.4%) | 144(100.0%) | 110(98.2%) | 139(98.6%) |  |
| Yes | 5(0.6 %) | 0(0.0%) | 1(0.6%) | 0(0.0%) | 2(1.8%) | 2(1.4%) |  |
| Diabetes Mellitus (DM) | | | | | | | |
| TNM I-III | 2237 | 593(26.5%) | 494(22.1%) | 400(17.9%) | 359(16.0%) | 391(17.5%) | 0.061 |
| No | 1878(84.0%) | 483(81.5%) | 408(82.6%) | 335(83.8%) | 316(88.0%) | 336(85.9%) |  |
| Yes | 359(16.0%) | 110(18.5%) | 86(17.4%) | 65(16.3%) | 43(12.0%) | 55(14.1%) |  |
| TNM III | 779 | 215(27.6%) | 167(21.4%) | 144(18.5%) | 112(14.4%) | 141(18.1%) | 0.418 |
| No | 636(81.6%) | 170(79.1%) | 134(80.2%) | 117(81.3%) | 98(87.5%) | 117(83.0%) |  |
| Yes | 143(18.4%) | 45(20.9%) | 33(19.8%) | 27(18.8%) | 14(12.5%) | 24(17.0%) |  |
| Hemiparaplegia | | | | | | | |
| TNM I-III | 2236 | 592(26.5%) | 494(22.1%) | 400(17.9%) | 359(16.1%) | 391(17.5%) | 0.385 |
| No | 2230(99.7%) | 589(99.5%) | 493(99.8%) | 398(99.5%) | 359(100.0%) | 391(100.0%) |  |
| Yes | 6(0.2%) | 3(0.5%) | 1(0.2%) | 2(0.5%) | 0(0.0%) | 0(0.0%) |  |
| TNM III | 779 | 215(27.6%) | 167(21.4%) | 144(18.5%) | 112(14.4%) | 141(18.1%) | 0.708 |
| No | 775(99.5%) | 213(99.1%) | 166(99.4%) | 143(99.3%) | 112(100.0%) | 141(100.0%) |  |
| Yes | 4(0.5%) | 2(0.9%) | 1(0.6%) | 1(0.7%) | 0(0.0%) | 0(0.0%) |  |
| HIV | | | | | | | |
| TNM I-III | 2236 | 592(26.5%) | 494(22.1%) | 400(17.9%) | 359(16.1%) | 391(17.5%) | x |
| No | 2236(100.0%) | 592(100.0%) | 494(100.0%) | 400(100.0%) | 359(100.0%) | 391(100.0%) |  |
| Yes | 0(0.0%) | 0(0.0%) | 0(0.0%) | 0(0.0%) | 0(0.0%) | 0(0.0%) |  |
| TNM III | 779 | 215(27.6%) | 167(21.4%) | 144(18.5%) | 112(14.4%) | 141(18.1%) | X |
| No | 779(100.0%) | 215(100.0%) | 167(100.0%) | 144(100.0%) | 112(100.0%) | 141(100.0%) |  |
| Yes | 0(0.0%) | 0(0.0%) | 0(0.0%) | 0(0.0%) | 0(0.0%) | 0(0.0%) |  |
| Liver disease | | | | | | | |
| TNM I-III | 2236 | 592(26.5%) | 494(22.1%) | 400(17.9%) | 359(16.1%) | 391(17.5%) | 0.184 |
| No | 2217(99.2%) | 585(98.8%) | 491(99.4%) | 398(99.5%) | 353(98.3%) | 390(100.0%) |  |
| Yes | 19(0.8%) | 7(1.2%) | 3(0.6%) | 2(0.5%) | 6(1.7%) | 1(0.0%) |  |
| TNM III | 779 | 215(27.6%) | 167(21.4%) | 144(18.5%) | 112(14.4%) | 141(18.1%) | 0.335 |
| No | 769(98.7%) | 210(97.7%) | 164(98.2%) | 144(100.0%) | 111(99.1%) | 140(99.3%) |  |
| Yes | 10(1.3%) | 5(2.3%) | 3(1.8%) | 0(0.0%) | 1(0.9%) | 1(0.7%) |  |
| Lung disease | | | | | | | |
| TNM I-III | 2236 | 592(26.5%) | 494(22.1%) | 400(17.9%) | 359(16.1%) | 391(17.5%) | 0.020 |
| No | 1914(85.6%) | 490(82.8%) | 412(83.4%) | 350(87.5%) | 320(89.1%) | 342(87.5%) |  |
| Yes | 322(14.4%) | 102(17.2%) | 82(16.6%) | 50(12.5%) | 39(10.9%) | 49(12.5%) |  |
| TNM III | 779 | 215(27.6%) | 167(21.4%) | 144(18.5%) | 112(14.4%) | 141(18.1%) | 0.189 |
| No | 668(86.6%) | 180(83.7%) | 137(82.0%) | 129(89.6%) | 101(90.2%) | 121(85.8%) |  |
| Yes | 111(14.2%) | 35(16.3%) | 30(18.0%) | 15(10.4%) | 11(9.8%) | 20(14.2%) |  |
| Malignancy | | | | | | | |
| TNM I-III | 2237 | 593(26.5%) | 494(22.1%) | 400(17.9%) | 359(16.0%) | 391(17.5%) | 0.193 |
| No | 2017(90.2%) | 536(90.4%) | 457(92.5%) | 356(89.0%) | 315(87.7%) | 353(90.3%) |  |
| Yes | 220(9.8%) | 57(9.6%) | 37(7.5%) | 44(11.0%) | 44(12.3%) | 38(9.7%) |  |
| TNM III | 779 | 215(27.6%) | 167(21.4%) | 144(18.5%) | 112(14.4%) | 141(18.1%) | 0.126 |
| No | 713(91.5%) | 202(94.0%) | 155(92.8%) | 125(86.8%) | 100(89.3%) | 131(93.0%) |  |
| Yes | 66(8.5%) | 13(6.0%) | 12(7.2%) | 19(13.2%) | 12(10.7%) | 10(7.0%) |  |
| Metastases in solid organ | | | | | | | |
| TNM I-III | 2236 | 592(26.5%) | 494(22.1%) | 400(17.9%) | 359(16.1%) | 391(17.5%) | 0.332 |
| No | 2235(100.0%) | 592(100.0%) | 494(100.0%) | 399(99.8%) | 359(100.0%) | 391(100.0%) |  |
| Yes | 1(0.0%) | 0(0.0%) | 0(0.0%) | 1(0.3%) | 0(0.0%) | 0(0.0%) |  |
| TNM III | 779 | 215(27.6%) | 167(21.4%) | 144(18.5%) | 112(14.4%) | 141(18.1%) | 0.353 |
| No | 778(99.9%) | 215(100.0%) | 167(100.0%) | 143(99.3%) | 112(100.0%) | 141(100.0%) |  |
| Yes | 1(0.1%) | 0(0.0%) | 0(0.0%) | 1(0.7%) | 0(0.0%) | 0(0.0%) |  |
| Myocardial infarction(MI) | | | | | | | |
| TNM I-III | 2237 | 592(26.5%) | 494(22.1%) | 401(17.9%) | 359(16.0%) | 391(17.5%) | 0.007 |
| No | 2097(93.7%) | 539(91.0%) | 459(92.9%) | 381(95.0%) | 343(95.5%) | 375(96.0%) |  |
| Yes | 140(6.3%) | 53(9.0%) | 35(7.1%) | 20(5.0%) | 16(4.5%) | 16(4.1%) |  |
| TNM III | 780 | 215(27.6%) | 167(21.4%) | 145(18.6%) | 112(14.4%) | 141(18.1%) | 0.015 |
| No | 736(94.4%) | 193(89.8%) | 162(97.0%) | 139(95.9%) | 108(96.4%) | 134(95.0%) |  |
| Yes | 44(5.6%) | 22(10.2%) | 5(3.0%) | 6(4.1%) | 4(3.6%) | 7(5.0%) |  |
| Peripheral Vascular disease (PVD) | | | | | | | |
| TNM I-III | 2236 | 592(26.5%) | 494(22.1%) | 400(17.9%) | 359(16.1%) | 391(17.5%) | 0.012 |
| No | 2172(97.1%) | 567(95.8%) | 474(96.0%) | 395(98.8%) | 353(98.3%) | 383(98.0%) |  |
| Yes | 64(2.9%) | 25(4.2%) | 20(4.0%) | 5(1.3%) | 6(1.7%) | 8(2.0%) |  |
| TNM III | 779 | 215(27.6%) | 167(21.4%) | 144(18.5%) | 112(14.4%) | 141(18.1%) | 0.036 |
| No | 760(97.6%) | 206(95.8%) | 160(95.8%) | 144(100.0%) | 111(99.1%) | 139(98.6%) |  |
| Yes | 19(2.4%) | 9(4.2%) | 7(4.2%) | 0(0.0%) | 1(0.9%) | 2(1.4%) |  |
| Renal Disease | | | | | | | |
| TNM I-III | 2236 | 592(26.5%) | 494(22.1%) | 400(17.9%) | 359(16.1%) | 391(17.5%) | 0.154 |
| No | 1998(89.4%) | 518(87.5%) | 440(89.1%) | 359(90.0%) | 333(92.8%) | 348(89.0%) |  |
| Yes | 238(10.6%) | 74(12.5%) | 54(10.9%) | 41(10.3%) | 26(7.2%) | 43(11.0%) |  |
| TNM III | 779 | 215(27.6%) | 167(21.4%) | 144(18.5%) | 112(14.4%) | 141(18.1%) | 0.740 |
| No | 689(88.4%) | 191(88.8%) | 144(86.2%) | 129(89.3%) | 102(91.1%) | 123(87.2%) |  |
| Yes | 90(11.6%) | 24(11.2%) | 23(13.8%) | 15(10.4%) | 10(8.9%) | 18(12.8%) |  |
| Rheumatological disease | | | | | | | |
| TNM I-III | 2236 | 592(26.5%) | 494(22.1%) | 400(17.9%) | 359(16.1%) | 391(17.5%) | 0.705 |
| No | 2200(98.4%) | 581(98.1%) | 484(98.0%) | 394(98.5%) | 356(99.2%) | 385(98.5%) |  |
| Yes | 36(1.6%) | 11(1.9%) | 10(2.0%) | 6(1.5%) | 3(0.8%) | 6(1.5%) |  |
| TNM III | 779 | 215(27.6%) | 167(21.4%) | 144(18.5%) | 112(14.4%) | 141(18.1%) | 0.644 |
| No | 767(98.5%) | 210(97.7%) | 166(99.4%) | 142(98.6%) | 111(99.1%) | 138(97.9%) |  |
| Yes | 12(1.5%) | 5(2.3%) | 1(0.6%) | 2(1.4%) | 1(1.4%) | 3(2.1%) |  |

Supplementary Table 2- Association between clinicopathological factors and CSS for TNM I-III and TNM stage III-univariable and multivariable analysis (CSS).

| Variable | TNM I-III | | | | TNM III | | | |
| --- | --- | --- | --- | --- | --- | --- | --- | --- |
|  | Univariable Analysis | | Multivariable Analysis | | Univariable Analysis | | Multivariable Analysis | |
|  | HR  (95% CI) | p | HR  (95% CI) | p | HR  (95% CI) | p | HR  (95% CI) | p |
| SIMD | - | 0.179 | - | - | - | 0.239 | - | - |
| ASA | 1.26  (1.02-1.55) | 0.032 | - | 0.298 | 1.30  (1.02-1.65) | 0.034 | - | 0.609 |
| Smoking | - | 0.820 | - | - | - | 0.963 | - | - |
| Body Mass Index (BMI) | - | 0.154 | - | - | - | 0.329 | - | - |
| CRP/Albumin ratio (CAR) | 1.21  (1.08-1.35) | <0.001 | - | 0.108 | 1.24  (1.08-1.43) | 0.003 | 1.24  (1.07-1.44) | 0.004 |
| Diabetes Mellitus | - | 0.311 | - | - |  |  |  |  |
| Lung disease | - | 0.909 | - | - |  |  |  |  |
| Myocardial infarction | - | 0.145 | - | - | 1.88  (1.01-3.47) | 0.045 | 2.19  (1.00-4.78) | 0.049 |
| Peripheral Vascular Disease | - | 0.788 | - | - | - | 0.319 | - | - |
| Receipt of Adjuvant Chemotherapy |  |  |  |  | 0.60  (0.42-0.85) | 0.004 | 0.46  (0.29-0.73) | <0.001 |
| T stage | 3.41  (2.74-4.25) | <0.001 | 2.47  (1.82-3.34) | <0.001 |  |  |  |  |
| EVMI | 3.31  (2.48-4.42) | <0.001 | 1.90  (1.27-2.84) | 0.002 |  |  |  |  |
